# iTRAQ-Based Proteomic Profile Analysis of the Hepatopancreas of Caribbean Spiny Lobsters Infected With *Panulirus argus* Virus 1: Metabolic and Physiological Implications

**DOI:** 10.3389/fmicb.2020.01084

**Published:** 2020-05-29

**Authors:** Jesús Alejandro Zamora-Briseño, Eliel Ruiz-May, José Miguel Elizalde-Contreras, Ioreni Margarita Hernández-Velázquez, Ariadne Hernández-Pérez, Ana Guadalupe Fuentes-García, Nancy Herrera-Salvatierra, Patricia Briones-Fourzán, Cristina Pascual-Jiménez, Enrique Lozano-Álvarez, Rossanna Rodríguez-Canul

**Affiliations:** ^1^Laboratorio de Inmunología y Biología Molecular, Departamento de Recursos del Mar, Centro de Investigación y de Estudios Avanzados del Instituto Politécnico Nacional-Unidad Mérida, Mérida, Mexico; ^2^Instituto de Ecología, Red de Estudios Moleculares Avanzados, Clúster Científico y Tecnológico BioMimic^®^, Xalapa, Mexico; ^3^Department of Comparative Physiology, Uppsala University, Norbyvägen, Sweden; ^4^Unidad Académica de Sistemas Arrecifales, Instituto de Ciencias del Mar y Limnología, Universidad Nacional Autónoma de México, Puerto Morelos, Mexico; ^5^Unidad Multidisciplinaria de Docencia e Investigación, Facultad de Ciencias, Universidad Nacional Autónoma de México, Sisal, Mexico

**Keywords:** PaV1, hepatopancreas, viral infection, proteomics, *Panulirus argus*

## Abstract

The Caribbean spiny lobster *Panulirus argus* (Latreille, 1084) sustains economically valuable fisheries throughout the wider Caribbean region. This species is currently affected by the pathogenic virus *Panulirus argus* Virus 1 (PaV1) that causes a systemic and chronic-degenerative infection in juvenile spiny lobsters *P. argus*. To date, there is no available information regarding the host alterations induced by this pathogen at the molecular level. In the present study, comparative proteomic analyses of the changes in the hepatopancreas between infected and non-infected juvenile lobsters were analyzed by isobaric tags for relative and absolute quantitation (iTRAQ) coupled to synchronous precursor selection (SPS)-based MS^3^. We identified a total of 636 proteins, being 68 down-regulated and 71 up-regulated proteins. Among the down-regulated proteins, we identified several enzymes involved in the metabolism of hormones and lipids, digestive proteases and glycosidases, while proteins associated with the histone core, protein synthesis, immune response and RNA regulation were up-regulated. Several misregulated enzymes involved in the regulation of neuromodulators were also identified. RT-qPCR assays were used to validate the expression of transcripts encoding for selected differential proteins that were in concordance to proteomic data, as well as the tendency observed in the enzymatic activities of trypsin, chymotrypsin, and glycosidase. In a similar way, we observed glycogen reduction in muscle, and an increase in plasma acylglycerides and glucose, which may be explained by proteomic data. This study provides the first insight into the molecular changes in the hepatopancreas of Caribbean spiny lobsters associated to PaV1 infection. Data provided herein would help to clarify the origin of the molecular misregulations observed at macroscopic level in this host-pathogen interaction.

## Introduction

The Caribbean spiny lobster *Panulirus argus* (Latreille, 1084) sustains economically valuable fisheries throughout all the Great Caribbean region (Goldstein et al., 2008; Ehrhardt et al., 2019). The distribution of this species encompasses from North Carolina to Venezuela, including the wider Caribbean Sea region (Carpenter and Niem, 2001; Williams, 2007). In Mexico, *P. argus* is captured mainly in the Yucatan Peninsula, along the coasts of Yucatan and Quintana Roo (FAO, 1998).

This species is currently affected by the pathogenic virus *Panulirus argus* Virus 1 (PaV1) (Shields and Behringer, 2004; Huchin-Mian et al., 2008). PaV1 is a large, non-enveloped, icosahedral DNA virus with a nucleocapsid approximately 187 nm in size (Shields and Behringer, 2004; Behringer et al., 2011), and is the only known viral agent that specifically infects *P. argus* in their natural environment (Behringer et al., 2011). PaV1 has been classified recently within a new family called *Mininucleoviridae*, because its replication occurs in the nucleus and not in the cytoplasm of infected cells (Subramaniam et al., 2020).

Although the virus has been reported in adults, sub-adults, juveniles and pueruli (Li et al., 2008; Huchin-Mian et al., 2008, 2009, 2013; Cruz-Quintana et al., 2011; Lozano-Álvarez et al., 2015), PaV1 infection shows a strong ontogenic component, with juvenile lobsters being more susceptible to infection than the other stages (Shields and Behringer, 2004; Lozano-Álvarez et al., 2008). In juvenile lobsters, PaV1 produces a chronic-degenerative infection that alters the physiology and behavior of severely infected organisms (Herrera-Salvatierra et al., 2019).

Since its discovery in 2000, PaV1 has been studied due to its potential danger to wild populations of lobsters given its high prevalence and mortalities rates in juvenile lobsters (15–45 mm carapace length, CL) (Huchin-Mian et al., 2008; Lozano-Álvarez et al., 2008; Candia-Zulbarán et al., 2012; Moss et al., 2013), for which mortalities of up to 100% have been recorded under experimental conditions (Li et al., 2008).

The primary sites of PaV1 infection are the fixed phagocytes of the hepatopancreas and the hyaline and semigranular hemocytes, followed by the soft connective tissue surrounding the hepatopancreas tubules (Shields and Behringer, 2004; Li et al., 2008). In advanced stages of infection, the virus spreads into the gills, heart, intestine, nerve cells, and cuticular epidermis. Hemolymph’s appearance becomes milky in and loses its coagulation abilities (Shields and Behringer, 2004; Li and Shields, 2007; Li et al., 2008). Exoskeleton develops a reddish coloration, and lobsters exhibit behavioral changes; they stop feeding, become lethargic, cease grooming and foraging and diminish their capability of self-defense (Shields and Behringer, 2004; Li et al., 2008; Huchin-Mian et al., 2008, 2009; Herrera-Salvatierra et al., 2019). The presence of clinical signs (milky hemolymph, lethargy, reddish exoskeleton, carapace fouling) has been used to identify PaV1-infected lobsters in the field (Behringer et al., 2011; Candia-Zulbarán et al., 2012). Laboratory diagnostic methods include the identification and quantification of viral inclusions by histology (Shields and Behringer, 2004), fluorescent in-situ hybridization tests (Li et al., 2006), and PCR assays for the specific amplification of viral DNA fragments (Montgomery-Fullerton et al., 2007; Clark et al., 2018).

In crustaceans, the hepatopancreas is the organ where the digestion, absorption and storage of nutrients occurs, and from which the nutrients are transported to all the tissues (Barker and Gibson, 1977; Jiang et al., 2009). In this organ, lobsters store a limited amount of energy reserves, particularly lipids and glycogen inclusions, that are used during molting, fasting and reproduction (Battison et al., 2014). During PaV1 infection, energy reserves decreases as severity of infection increases (Li et al., 2008; Pascual-Jiménez et al., 2012), In the last stage of PaV1 infection, the hepatopancreas becomes highly damaged (Li et al., 2008; Huchin-Mian et al., 2013), and the immunological and physiological responses of infected lobsters become highly compromised (Pascual-Jiménez et al., 2012; Herrera-Salvatierra et al., 2019). Despite the importance of the hepatopancreas in this pathosystem, the information about the proteins deregulated in this organ during PaV1 infection is almost null. Knowing the changes produced by PaV1 at the protein level would help to elucidate the biochemical imbalances produced in the hepatopancreas, which could be useful to understand the physiological alterations occurring in infected lobsters.

The aims of this study were to examine the changes occurring in the proteome of the hepatopancreas of juvenile spiny lobsters infected with PaV1, and to validate this proteomic profile through the determination of some physiological responses of PaV1 infected and non-infected lobsters, and finally, to propose several action modes by which PaV1 produces the observed physiological alterations in infected lobsters. Results of this study will help to elucidate the molecular mechanisms affected during PaV1 infection in juveniles *P. argus*.

## Materials and Methods

**FIGURE 1:**
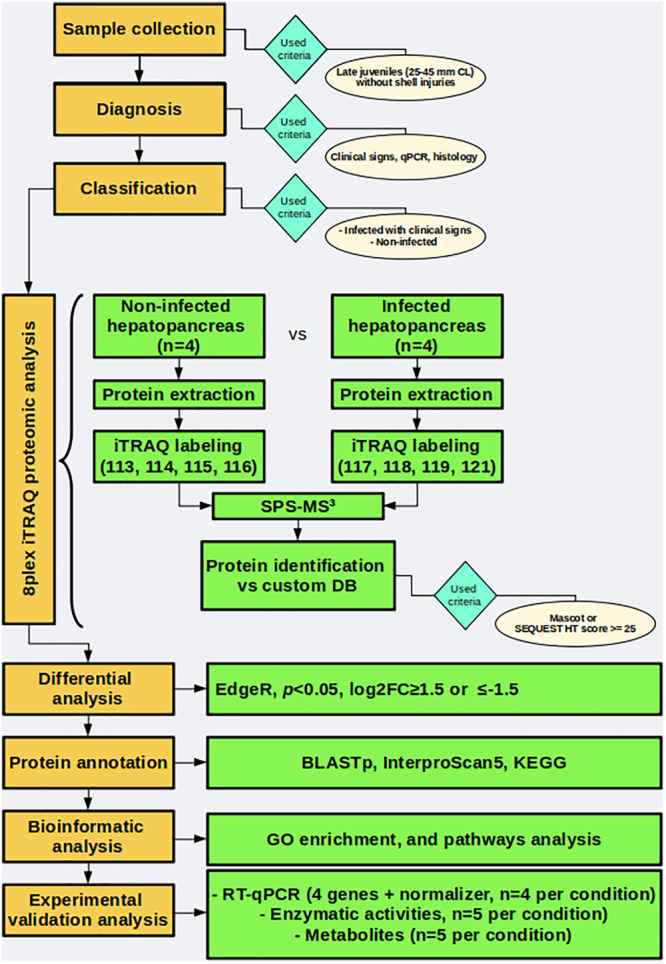
General workflow followed in this work.

Naturally infected and uninfected wild spiny lobsters were used to evaluate changes induced by PaV1 in the proteome of the hepatopancreas. A general workflow used herein is shown in Figure 1.

### Sample Collection and Histological Analysis

Seventy-two live juvenile lobsters were caught by SCUBA diving at the Puerto Morelos reef lagoon, in a natural protected area in Mexico (centered at 20° 51′ N, 86° 52′ W) in November 2017. This reef lagoon is a nursery habitat for juveniles of *P. argus*, where a relatively high prevalence of PaV1 has been detected (Briones-Fourzàn and Lozano-Álvarez, 2001; Huchin-Mian et al., 2008, 2009).

Lobsters were transported to the laboratory in aerated plastic containers within 1 h of capture. They were sexed, measured (carapace length, CL, in mm) with a Vernier caliper, examined for injuries and clinical signs of PaV1 infection (milky hemolymph and carapace reddish coloration), and molt staged.

Hemolymph was collected from the pericardial sinus of each spiny lobster using a chilled syringe needle. The area was previously sterilized with 70% ethanol. Hemolymph was diluted in two volumes of cooled anticoagulant solution (Vargas-Albores et al., 1993). Total hemocytes were removed by centrifugation at 5,000 × *g* during 5 min at 4°C and plasma was recovered for estimation of glucose and acilglycerides concentration in plasma (Pascual-Jiménez et al., 2012; Herrera-Salvatierra et al., 2019).

Lobsters were categorized as positive and negative samples, based on clinical signs (i.e. milky hemolymph and reddish coloration of the shell) (Shields and Behringer, 2004; Behringer et al., 2011), presence of eosinophilic viral inclusion in the hepatopancreas (Li et al., 2008; Huchin-Mian et al., 2008, 2009), and viral load (Clark et al., 2018).

Lobsters were then anesthetized by immersion in cold sea water (4 °C for 5 min) and euthanized by cutting the ventral nerve cord. Only late juveniles (25–45 mm CL) in intermolt were used (Herrnkind, 1980). A portion of the hepatopancreas (≈ 200 mg) was collected and placed in histology cassettes. Samples were fixed for 24 h in Davidson’s solution (22% formaldehyde, 33% ethanol, 11.5% glacial acetic acid and 33.5% distilled water), and then transferred into a 70% ethanol solution until processed. Routine histology procedure was performed including dehydration, embedding, and staining (HandE) on 5 μm cross-section cuts. The grade of viral infection was assessed according to the categorical scale (0–4) proposed previously (Li et al., 2008). Additionally, 100 mg of hepatopancreas tissue were fixed in 96% ethanol for gDNA isolation, 1000 mg of hepatopancreas of each sample was frozen in liquid nitrogen for quantification of enzymatic activity and metabolites and maintained at −70°C until further analyses. Finally, the remaining samples of hepatopancreas were fixed and homogenized in five volumes of RNA Later^®^ solution (Thermo Fisher©) and preserved in liquid nitrogen for protein and RNA extraction.

### PaV1 Quantification by qPCR

Viral load quantification by qPCR in hepatopancreas was performed, according to Clark et al. (2018), with minor modifications. For this, 100 mg of hepatopancreas of each sample was extracted using the Quick-DNA^TM^ Universal Kit (Zymo Research©). gDNA from an infected sample was used to amplify a 499 bp region of the PaV1 (Montgomery-Fullerton et al., 2007). Amplicon was cloned into the pJET1.2 plasmid using the CloneJET PCR Cloning Kit^TM^ (Thermo Scientific©). Insert was sequenced and plasmid was used for further analysis. A TaqMan probe was used to detect part of this region. In order to quantify viral load, standard curves were carried out using a serial dilution (1:5) of the recombinant plasmid equivalent to a dynamic range from 30 to 3 × 10^9^ PaV1 copies. qPCRs were performed in a Rotor Gen^®^ real-time PCR detection system in a final volume of 10 μl, with 5 μl QuantiNova Probe PCR (QIAGEN©), 0.15 μM of each primer, 0.3 μl of 20X PaV1-TaqMan and 2 μl of plasmid dilution or 2 μl (65–140 ng) of gDNA. Cycling conditions were 10 min at 95°C, followed by 40 cycles of 15 s at 95°C and 1 min at 62°C.

**FIGURE 2:**
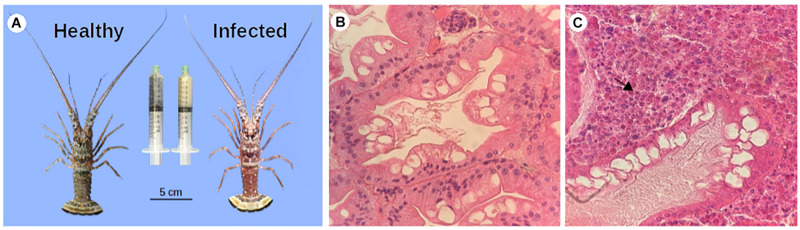
PaV1 infected lobsters vs. uninfected lobsters. **(A)** Macroscopic signs: a reddish exoskeleton and milky hemolymph (right syringe) were observed in infected lobsters with a very high viral load. Histopathology of hepatopancreas of healthy **(B)**, and infected **(C)** lobsters. Hepatopancreas sections were dissected, fixed and stained with H & E and observed at 40X. The viral particles are stacked inside the sample (arrow).

Based on these results, lobsters were allotted as positive and negative for PaV1 infection. These groups were considered herein as infected and healthy lobsters, and used in further analyses (Figure 2).

### Protein Extraction for Proteomic Analysis

The total crude protein extract was prepared from 250 mg of hepatopancreas of each sample, using the SDS-phenol extraction protocol (Faurobert et al., 2006), with minor modifications. Briefly, samples were macerated in 3 mL of extraction buffer (500 mM Tris–HCl, pH 8.0, 50 mM EDTA, 700 mM sucrose, 100 mM KCl, 2% β-mercaptoethanol, and freshly prepared 4 mM phenylmethylsulfonyl fluoride), vortexed, and incubated by shaking for 10 min on ice. Afterward, an equal volume of Tris-buffered phenol (pH 8.0) was added, and the mixture was incubated on a shaker for 10 min at room temperature. Samples were centrifuged for 10 min at 5,500 × *g* and 4°C. The phenolic upper phase was recovered carefully to avoid contact with the interphase and poured into a new tube. Then, four volumes of cooled precipitation solution (100 mM sodium acetate in methanol) were added and the samples were stored overnight at −20°C. Proteins were centrifuged for 10 min, 5,500 × *g* at 4°C and washed with cooled precipitation solution, then with cooled 80% acetone, and finally with 70% ethanol. After each washing step, samples were centrifuged for 5 min at 5,500 × *g* and 4°C. Finally, the proteins were dried at room temperature. The protein pellet was resuspended in solubilization buffer (1% SDS in phosphate buffer, 100 mM, pH 7.5) and quantified using a micro BCA assay kit (Thermo Scientific^®^).

### Proteomic Analysis

The proteomic pipeline, including protein reduction, alkylation, digestion, desalting, and nano LC-MS/MS analysis, was performed as previously reported (Hernández-Pérez et al., 2019), with minor modifications. Comparative proteomics was carried out with Isobaric tags for relative and absolute quantitation (iTRAQ) and synchronous precursor selection-MS^3^ in an UltiMate 3000 RSLC system (Dionex©) coupled to an Orbitrap Fusion Tribrid (Thermo-Fisher Scientific©) mass spectrometer equipped with an “EASY Spray” nano ion source (Thermo-Fisher Scientific©). iTRAQ 8-plex reagents (cat. number 4381663, Sigma-Aldrich©) with varying molecular weights were applied as isobaric labels for the comparative quantification of proteins between the digested samples: 113, 114, 115, and 116 for the healthy group and 117, 118, 119, and 120 for the infected group, according to the manufacturer’s protocol.

### Bioinformatic Analysis

For the proteomic analysis a custom database was prepared using a set of predicted proteins from a reference transcriptome from the hepatopancreas of *P. argus* previously obtained (data not shown) using TransDecoder^1^. These predicted sequences were added to the sequences downloaded from UniProt (Bateman et al., 2017) of the Arthropoda group. Total sequences were filtered with CD-HIT program (with -c 0.98 and -n 5 as parameters) to reduce redundancies (Li and Godzik, 2006). The raw data was processed with Proteome Discoverer 2.1 (PD, Thermo Fisher Scientific©). The subsequent searches were carried out using Mascot search engine (version 2.4.1, Matrix Science©), and SEQUEST HT (Eng et al., 1994). We considered a 25 score for protein identification.

We considered as search parameters the full-tryptic protease specificity, two missed cleavage, and carbamidomethylation of cysteine (+57.021 Da), iTRAQ 8-plex tagged (+229.163 Da) in N-terminal and lysine residues (K) as static modifications. We also considered methionine oxidation (+15.995 Da) and deamidation in asparagine/glutamine (+0.984 Da) as dynamic modifications. Protein identification was carried out at lower resolution in the linear ion trap with tolerances of ±10 ppm and ±0.6 Da. Peptide hits were filtered for a maximum of 1% FDR using the Percolator algorithm (Käll et al., 2007).

A principal component analysis of the abundance of the proteins in each sample was constructed with ClustVis (Metsalu and Vilo, 2015), as well as a heatmap of the differential proteins. Differential proteins were determined using the Bioconductor EdgeR 2.14 package. We defined differential regulated proteins as those with a log2 fold change (FC) ≤ −1.5 or >1.5 and a *p*-value < 0.05 in order to select the most altered proteins. With this tool, we also performed Pearson’s correlation analysis to evaluate the level of dispersion of the total protein abundance data per condition. For the annotation of all identified proteins, local BLASTp searches against Nr and Uniprot for invertebrate databases were performed. In addition, InterproScan5 (Jones et al., 2014) was used to obtain the GO ontologies and pFAM searches. Gene ontology enrichment analysis was performed using Fisher’s *T*-test in Blast2GO (Conesa et al., 2005) with a *p*-value of 0.05 as the threshold, and the relative abundance of each enriched category was plotted. Protein sequences were also annotated using Kyoto Encyclopedia of Genes and Genomes (KEGG) (Ogata et al., 2000). BlastKOALA (Kanehisa et al., 2016) was used to obtain the KEGG Ontology (KO) number of each sequence. The complete list of both up and down-regulated proteins was used to feed KEGG Mapper (Kanehisa and Sato, 2019) to reconstruct the different pathways associated to each KO number. All graphs were constructed with the ggplot2 package (Ginestet, 2011).

### Enzymatic Activity and Metabolites Quantification

To validate the proteomic results, we quantified the enzymatic activities of key digestive enzymes (trypsin, chymotrypsin and glycosidase) as well as the content of glycogen in hepatopancreas. For these determinations, the hepatopancreas was homogenized with water at a 1:6 dilution (W: V) and centrifuged at 4°C for 20 min at 16 000 × *g*, and the supernatant was used for the assays. All determinations were run in duplicates using five samples per condition. The protein content of hepatopancreas extracts was measured following the Bradford assay using Bovine Serum Albumine (BSA) as the standard. Enzyme activities were expressed as specific activity (U⋅g^–1^ of total protein).

#### Trypsin Activity

Trypsin activity was measured using a 96-well microplate. 5 μL of enzyme extract were mixed with 290 μL of Tris buffer (100 mM, pH 8) and 6 μL of 100 mM N-benzoyl-DLarginine p-nitroanilide (BapNA) disolved in dimethyl sulfoxide (DMSO). The reaction mix was incubated at 60°C. Chymotrypsin-like activity was measured with 100 mM Suc-Ala-Ala-Pro-Phe-p-nitroanilide (SApNA). 5 μL of enzyme extract were mixed with 290 μL of Tris buffer (100 mM, pH 7), and 6 μL of substrate solutions prepared in DMSO. The mixture was incubated at 50°C. For each enzyme, absorbance was recorded at 405 nm.

#### Glycosidase Activity

Glycosidase activity was measured with 120 mM p-Nitrophenil D-GlycoPyranoside (PNPG) as substrate in a 96-well microplate. For this, 5 μL of enzyme extract were mixed with 145 μL sodium phosphate buffer (50 mM, pH 6 at 60°C) and 6 μL of a substrate solution prepared in dimetylsulfoxide DMSO that were incubated at 60 °C for 60 min. 145 μL of sodium carbonate were used to stop the reaction. Absorbance was recorded at 405 nm.

#### Glycogen Determination

Glycogen was measured in 40 mg of hepatopancreas. Frozen tissue was homogenized with 5% of trichloroacetic acid (TCA), and centrifuged for 6 min at 5,000 × *g*. 100 μL of the supernatants were mixed with 500 μL ethanol 95% and incubated at 37 °C for 3 h; the mix was centrifuged for 15 min at 5,000 × *g* and the pellet was dissolved in 20 μL of distilled water at 100°C, 200 μL of phenol 5% and 1 mL of sulfuric acid. Absorbance was recorded at 490 nm. Concentration was reported as mg⋅g^–1^, calculated from a commercial glucose standard reagent (1 mg⋅ml^–1^) (Sera Pak Plus^®^).

In addition, glucose, and acylglycerids concentrations in plasma were determined using a clinical diagnostic reactive kit (Sera Pak Plus^®^; Bayer, Whippany, NJ, United States). Concentrations were reported as mg⋅mL^–1^.

Kruskal–Wallis non-parametric analyses were used to test for statistically significant differences in each variable among healthy and infected lobsters. Statistical analysis were carried out in R (R Development Core Team, 2016).

### Proteomic Data Validation With RT-qPCR

To validate the inferred proteomic profile at the transcriptional level, four differentially regulated proteins were randomly chosen. Primers were designed using the program Primer3 Plus program (Untergasser et al., 2007), using the transcript sequences from a reference transcriptome of *P. argus* obtained previously (data not shown).

Total RNA was isolated from ∼100 mg hepatopancreas using the Direct-zol RNA^®^ protocol according to the manufacturer’s instructions. RNA integrity was checked in a 1% agarose gel. For cDNA synthesis, 1200 ng of total RNA were converted to cDNA using the RevertAid First Strand cDNA Synthesis Kit (Thermo Scientific^®^) following the manufacturer’s instruction. All the qPCRs were performed in a final volume of 10 μl, with 5 μl using the Luminaris HiGreen qPCR Master mix 2x (Thermo Scientific^®^), 0.3 μl of each primer (10 μM), and 1 μl of a 1:5 cDNA dilution as template. All qPCRs were performed in a Rotor Gene-Q detection system (QIAGEN^®^). Cycling conditions were as follows: UDG pre-treatment at 50 for 2 min, 10 min at 95°C, followed by 40 cycles of 15 s at 95°C, 30 s at 60°C, and 30 s at 72°C. In addition, melt curve analyses were performed to validate the amplicon specificity. The expression level of mRNA was normalized to the expression of pyruvate carboxylase gene (internal control). For each case, the fold of change was estimated as relative gene expression levels using the ddCt method (Livak and Schmittgen, 2001), transformed to log2 scale and compared with proteomic data.

## Results

### Sample Collection and Viral Quantification

Seventy-two juvenile spiny lobsters were captured and based on their viral load and clinical characteristics, we selected eight samples from each condition, grouped as infected and healthy. Mean ± SD carapace length (CL) was 37.9 ± 5.8 mm for the infected group and 33.4 ± 6.7 mm for the non-infected group. The lobsters that were classified as infected, clearly showed clinical signs of the disease, with a reddish carapace coloration and the hemolymph with a remarkable milky appearance (Figure 2A). This group had high viral loads by qPCR 5.22 ± 1.7 × 10^6^ (mean ± SD) viral copies ng^–1^ gDNA]. In contrast, in the uninfected group no clinical signs of the infection were observed, and viral loads were below the detection limit of the qPCR assay (<30 viral copies⋅μg^–1^ of gDNA).

In addition, histology of the hepatopancreas of infected lobsters showed a high number of eosinophilic viral inclusions (CAI) characterized by a structural damage, whereas in the non-infected group no damage was observed. Based on the categorical scale of Li et al. (2008), spiny lobsters in the infected group were categorized as heavily infected (grade 4), characterized by interstitial spaces of the hepatopancreas filled with numerous infected cells (>100 per section), atrophied hepatopancreatic tubules, and many infected cells present in spongy connective tissue (Figure 2C), in contrast to healthy samples (Figure 2B).

### Protein Identification and GO Annotation

Through our proteomic analysis, we detected isobaric tag signals that were related to 3487 identified peptides, 3059 of which were unique peptides that corresponded to 634 non-redundant proteins, which were detected and quantified in the infected and healthy samples (Supplementary Table S1). Supporting information was submitted to iProX (Ma et al., 2019), under the project number IPX0002128000.

**FIGURE 3:**
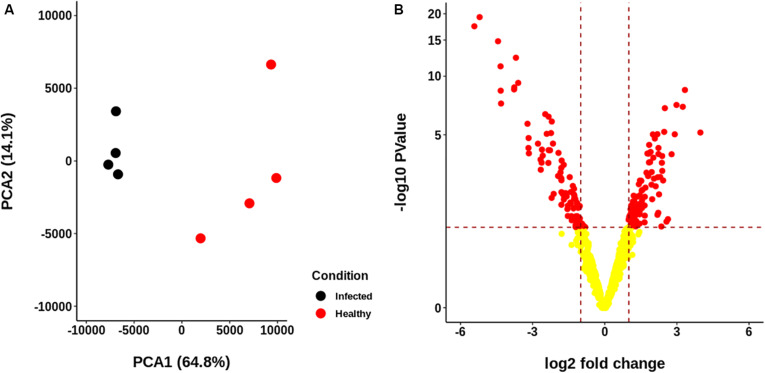
Descriptive statistics of the proteomic data generated for each sample group. **(A)** Principal Components Analysis (PCA) of the abundance of the identified proteome. No scaling was applied to rows; singular value decomposition (SVD) with imputation was used to calculate principal components. *X* and *Y* axis show principal component 1 and principal component 2, which explain 64.8 and 14.1% of the total variance, respectively. Healthy and infected samples are clearly separated. **(B)** Volcano plot of the abundances of the proteins identified. A total of 71 up-regulated and 68 down-regulated proteins were identified.

**FIGURE 4:**
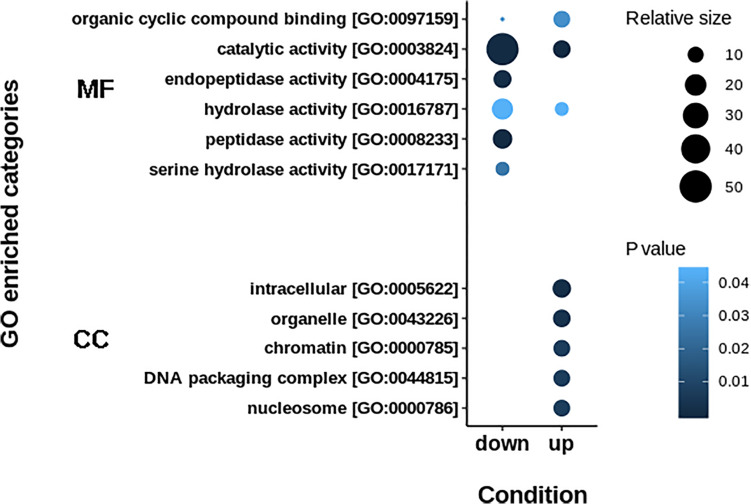
GO enrichment terms in up- and down-regulated proteins. Down-regulated proteins are principally implicated in catalytic activities, while up-regulated proteins are enriched in regulation of genetic information. MF: Molecular Function. CC: Cellular Component.

**FIGURE 5:**
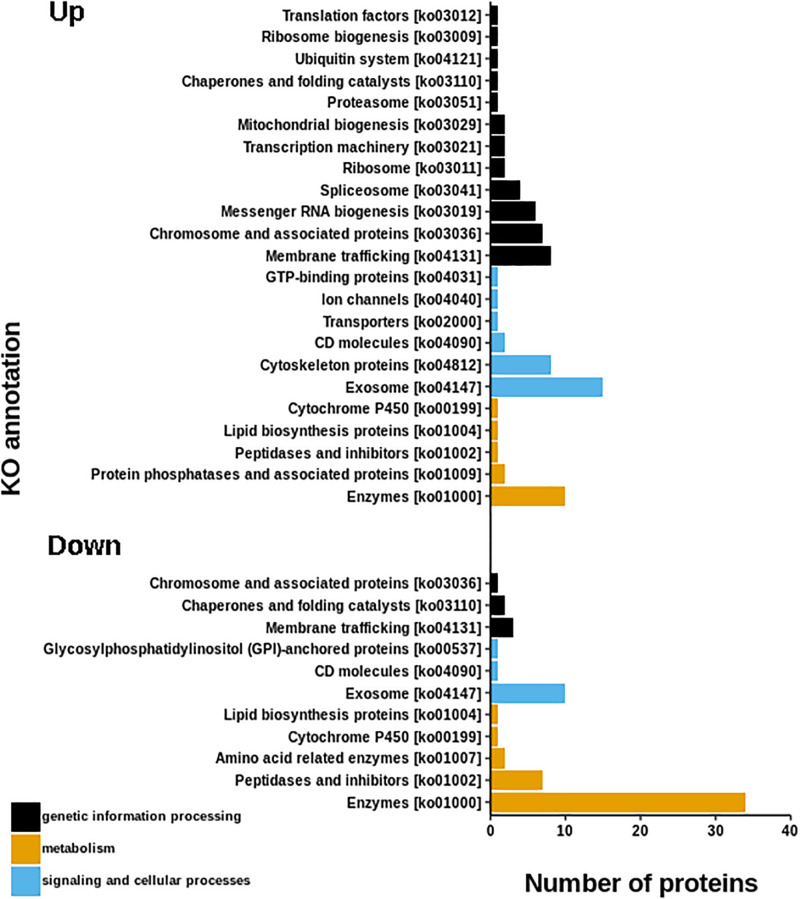
KEGG annotations of differential proteins. Graph shown that up-regulated proteins are more abundant in KO categories involved in genetic processing, while, down-regulated proteins are more abundant in metabolic process.

**FIGURE 6:**
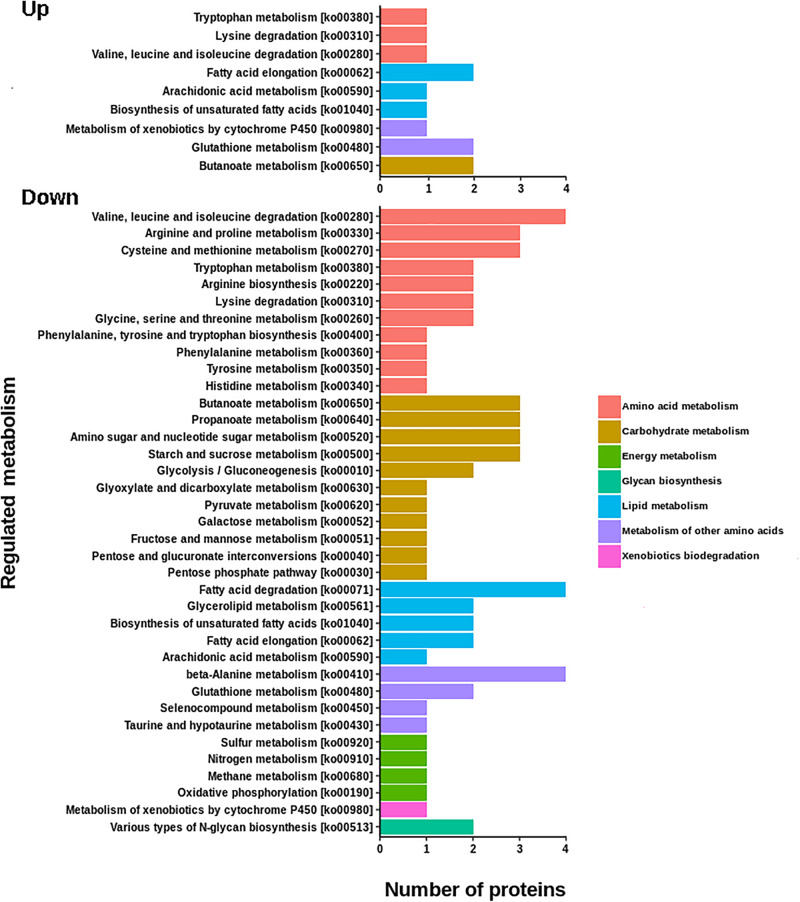
KEGG annotations of differential proteins involved in metabolic functions. The number of down regulated enzymes involved in metabolic process are almost three times more abundant that up-regulated enzymes. Down regulated proteins are associated to the metabolism of amino acids, carbohydrates, lipids, and energy, as well as with glycan biosynthesis.

The resulting proteome was distributed into 166 biological processes, 68 cellular components, and 259 molecular function categories. Among the biological processes, the more important are translational elongation, oxygen transport, lipid metabolism, microtubule-based process, carbohydrate metabolism, and immune response (Supplementary Figure S1). Although we were focused on the differential proteins, it is noteworthy that the molecular functions associated with the total proteome reflects the specialization of the hepatopancreas to metabolize nutrients according to the dietary composition. The diet of juvenile lobsters includes a high level of protein, with a low and moderate level of lipids and carbohydrates (Marx and Herrnkind, 1985; Cox et al., 1997; Johnston, 2003; Briones-Fourzán et al., 2003; Perera et al., 2012), which maintain a proportion with the ontologies distribution. The comparative proteomic analysis showed a clear differentiation of the proteins of the hepatopancreas between infected and non-infected samples (Figure 3A). Even more, Pearson’s correlation analysis showed that the total proteins are well clustered per each condition (Supplementary Figures S2B,C). From this analysis, 71 up-regulated proteins and 68 down-regulated proteins were identified (Figure 3B, Supplementary Figure S2A). The GO enrichment analysis showed that down-regulated proteins are enriched in molecular functions associated to enzymatic activities, while up-regulated proteins are associated to chromatin and packing and regulation of DNA (Figure 4). These results are in concordance with the annotations provided by KEGG. In this analysis, up-regulated proteins are clearly more abundant in KO categories involved in genetic processing. In contrast, down-regulated proteins are more abundant in processes associated to metabolism (Figure 5). Down regulated enzymes are almost three times more abundant than up-regulated enzymes. Among these enzymes, metabolism of amino acids, carbohydrates, lipids, and energy, as well as glycan biosynthesis categories are more abundant in down regulated proteins (Figure 6 and Supplementary Table S2).

### Down-Regulated Proteins

#### Proteases

In the down-regulated protein group there is a remarkable representation of digestive proteases. Within the set of repressed proteases, there are several key enzymes of the digestive machinery, such as meprin B [EC:3.4.24.63], trypsin 1 [EC:3.4.21.4] and trypsin 3 [EC:3.4.21.4], neprilysin [EC:3.4.24.11], legumain [EC:3.4.22.34], aminopeptidase N [EC:3.4.11.2], membrane dipeptidase [EC:3.4.13.19], Carboxypeptidase B [EC:3.4.17.-], chymotrypsin [EC:3.4.21.2], leucyl aminopeptidase [EC:3.4.11.1], and astacin [EC:3.4.24.21]. Some of these proteases are recognized as very important for the digestion and absorption of proteins ingested by the lobsters, and their repression results in an impairment in the digestion capabilities of proteins which represent the major component of the food intake of this species (Perera et al., 2012).

#### Enzymes Involved in Carbohydrate Metabolism

Three enzymes involved in carbohydrate metabolism appeared to be deregulated in the PaV1 infected group (Supplementary Figure S3). This was the case for beta-galactosidase [EC:3.2.1.23], a key provider in the production of energy and carbons through the breakdown of lactose to galactose and glucose. Another down-regulated enzyme was the UTP-glucose-1-phosphate uridylyltransferase [EC:2.7.7.9], a main component in the production of glycogen reserves. UTP-glucose-1-phosphate uridylyltransferase is involved in the biosynthesis and pyrophosphorolysis of UDP-glucose, the precursor used for glycogen and β-glucan biosynthesis (Silva-Sanchez et al., 2014). Another key down-regulated protein is the glycolytic enzyme fructose-bisphosphate aldolase [EC:4.1.2.13]. This enzyme occupies a central position in glycolysis and gluconeogenesis pathways (Ziveri et al., 2017). In the glycolysis, this enzyme is involved in step 4 of the sub-pathway that synthesizes D-glyceraldehyde 3-phosphate and glycerone phosphate from D-glucose.

Aldolase is also vital for the assembly of the Vacuolar H^+^-ATPases (V-ATPases) (Lu et al., 2004) which was also down-regulated. This is significant, since V-ATPase is primarily responsible for the establishment and maintenance of the acidic pH of endocytic and secretory organelles, pumping cytosolic H^+^ into their lumen in an ATP-dependent manner (Maxson and Grinstein, 2014).

#### Lipid Metabolism

The reconstructed pathway showed a dramatic impact of PaV1 on lipid metabolism (Supplementary Figure S4). For example, several enzymes involved in fatty acids degradation, such as enoyl-CoA hydratase/long-chain 3-hydroxyacyl-CoA dehydrogenase [EC:4.2.1.17, 1.1.1.211], acyl-CoA dehydrogenase [EC:1.3.8.7], acyl-CoA oxidase [EC:1.3.3.6], and aldehyde dehydrogenase (NAD+) [EC:1.2.1.3], were down regulated. Acyl-CoA oxidase, long-chain 3-hydroxyacyl-CoA dehydrogenase and acyl-CoA dehydrogenase plays an essential role in the mitochondrial beta-oxidation of fatty acids. On the other hand, the acyl-coenzyme A thioesterase 1/2/4 [EC:3.1.2.2], implicated in the biosynthesis of unsaturated fatty acids, was also repressed.

Similarly, the metabolism of triglycerides was also negatively affected. In this case, triacylglycerol lipase (TGL) [EC:3.1.1.3] was down regulated. As a consequence of the hierarchical regulation of lipolysis, TGL is a key limiting rate regulatory enzyme for this processes (Fredrikson et al., 1981; Watt and Spriet, 2010). Finally, mitochondrial aspartate aminotransferase [EC:2.6.1.1], an enzyme that participates in fat digestion and absorption, was also down-regulated.

### Up-Regulated Proteins

A generalized up-regulation of proteins involved in genetic control was observed. Within these proteins, the generalized up-regulation of H1, H2A, H2B, and H3 histone proteins is noteworthy (Supplementary Table S3). These proteins represent practically all the histone octamer (conformed of two copies of each of the histones H2A, H2B, H3, and H4), which are the core of the nucleosome around which DNA is tightly wrapped (Davey et al., 2002). Another up-regulated protein is the heterogeneous nuclear ribonucleoprotein A1/A3, which is involved in cytoplasmic trafficking of RNA, and pre-mRNA splicing (Ma et al., 2002).

We also identified several proteins associated with the phagosome structure and endocytosis, cytoskeleton regulation and organization, and intracellular trafficking, such as ankyrin, EH domain-containing protein 1, actin related protein 2/3 complex, subunit 1A/1B, F-actin, vacuolar protein sorting-associated protein (IST1), endocytosis actin beta/gamma 1, tubulin alpha (TUBA), and tubulin beta (TUBB), that were also up-regulated. Another up-regulated protein was cofilin, which is an actin-related identified protein. This protein depolymerizes F-actin at the slow-growing ends, creating new actin monomers for polymerization, and severs actin filaments, thus creating new barbed ends. Therefore, cofilin promotes the dynamics of F-actin networks (Niggli, 2014).

Up-regulation of several proteins involved in protein folding and processing in endoplasmic reticulum was also observed. These include the GTP-binding protein SAR1 [EC:3.6.5.-], transitional endoplasmic reticulum ATPase, molecular chaperone HtpG, and HSP90. These proteins are implicated in an important number of functions. For example, transitional endoplasmic reticulum ATPase regulates multiple processes including endoplasmic reticulum-associated degradation, ubiquitin-proteasome-dependent protein degradation, transcriptional control, cell cycle regulation, and DNA damage response (Zhang et al., 2015).

Finally, we identified the up-regulation of prophenol oxidase (proPO), which is a key component of the crustacean immune system and is activated against several pathogens (Cerenius and Soderhall, 2012).

### Deregulation of Proteins Involved in the Regulation and Biosynthesis of Hormones, Neurotransmitters and Neuromodulators

The down-regulation of relevant enzymes involved in hormonal regulation and development, such as the estrogen sulfotransferase (SULT1E) [EC:2.8.2.-] and the hemolymph juvenile hormone binding protein (JHBP) [EC:3.3.2.9], was also observed. Estrogen sulfotransferase is a cytosolic enzyme that catalyzes the sulfoconjugation and inactivation of estrogens (Tong et al., 2004), whereas JHBP is the carrier of the Juvenile hormone (JH). JH has a wide range of effects on the development of insects, for example stimulation of reproductive maturation. Therefore, the importance of JHBP lies in its capability to transport and protect the JH molecules from hydrolysis by non-specific esterases present in the haemolymph (Kołodziejczyk et al., 2003).

We also found several down-regulated proteins involved in the biosynthesis of neuromodulators and neurotransmitters. Among these enzymes, we found sepiapterin reductase [EC:1.1.1.153], which catalyzes the final reductions in tetra-hydrobiopterin (BH4) biosynthesis (Supplementary Figure S5). During these reactions, BH4 is converted to the quinonoid dihydrobiopterin, and is regenerated by sepiapterin reductase. BH4 is very important for the biosynthesis of neurotransmitters, since it is the cofactor of the phenylalanine hydroxylase [EC 1.14.16.1], tyrosine hydroxylase [EC 1.14.16.2], and tryptophan hydroxylase [EC 1.14.16.4], as well as nitric oxide synthase [EC 1.14.13.39] (Caccamo et al., 2013). These hydroxylases conform the family of biopterin-dependent aromatic amino acid hydroxylases, and catalyze the biosynthesis of neurotransmitters by hydroxylating aromatic amino acids (Kaufman, 2002). In this sense, phenylalanine hydroxylase [EC 1.14.16.1] hydroxylates phenylalanine to produce tyrosine (Patel et al., 2016), while tryptophan hydroxylase catalyzes the first and rate-limiting step in the biosynthesis of serotonin (Walther et al., 2003). Tyrosine hydroxylase catalyzes the conversion of tyrosine to L-DOPA. L-DOPA is a precursor for dopamine, which, in turn, is a precursor for important catecholamines such as noradrenaline and adrenaline (Daubner et al., 2011). Tyrosine hydroxylase catalyzes the rate limiting step in the synthesis of these compounds, and is the first enzyme in the synthesis of melatonin (Venero et al., 2002).

On the other hand, the down regulation of the sulfinoalanine decarboxylase (SAD) [EC:4.1.1.29] catalyzes the decarboxylation of L-aspartate, 3-sulfino-L-alanine (cysteine sulfinic acid), and L-cysteate to beta-alanine, hypotaurine and taurine, respectively (Supplementary Figure S6). This is relevant since taurine is a modulator of basic processes, such as osmotic pressure, cation homeostasis, enzyme activity, receptor regulation, cell development and cell signaling (Schaffer et al., 2010).

The glutamate metabolism is also affected by PaV1 infection. Glutamate, as well as glutamine and gamma-aminobutyric acid (GABA, an inhibitory neurotransmitter), represent essential amino acids for brain metabolism and function (Struzyéska and Sulkowski, 2004). We identified three enzymes involved in GABA and glutamine metabolism: succinate-semialdehyde dehydrogenase [EC:1.2.1.24], 4-aminobutyrate aminotransferase [EC:2.6.1.19 2.6.1.22], and glutamine synthetase [EC:6.3.1.2]. Both, 4-aminobutyrate aminotransferase and succinate-semialdehyde dehydrogenase are enzymes implicated in the catabolism of GABA (Supplementary Figure S7). The enzyme 4-aminobutyrate aminotransferase uses two substrates, GABA and 2-oxoglutarate to yield succinate semialdehyde and glutamate, respectively. Then, the former catalyzes the final step of the degradation of GABA, which oxidizes succinic semialdehyde to succinate coupled with NADH production (Moller et al., 2003), whereas glutamine synthase catalyzes the ATP-dependent condensation of glutamate with ammonia to generate glutamine (Liaw et al., 1995).

### Enzymatic Activity and Quantification of Metabolites and Transcripts to Validate the Proteomic Data

**FIGURE 7:**
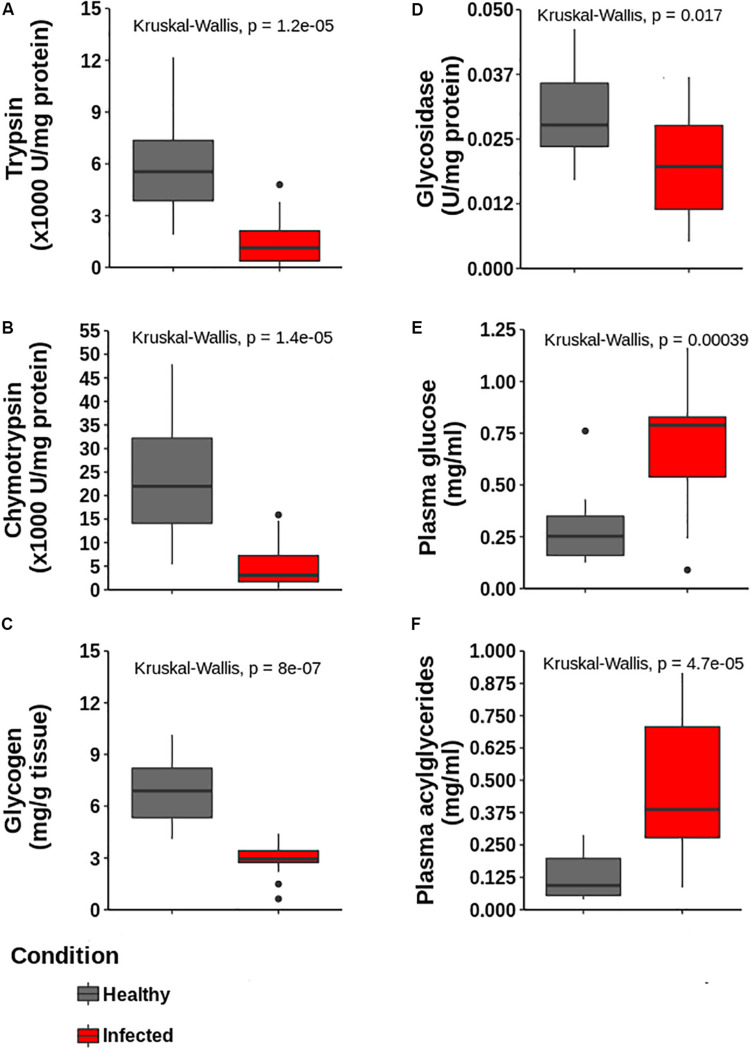
Determination of enzymatic activities and metabolites in hepatopancreas and plasma to validate proteomic data. **(A)** Trypsin, **(B)** Chymiotrypsin, **(C)** Glycogen content and **(D)** glycosidase activities in hepatopancreas. **(E,F)** Glucose and acylglycerides in plasma, respectively. All determinations are statistically different according to Kruskal–Wallis test (*p* < 0.05, *n* = 5 for each group).

The activity of the digestive enzymes chymotrypsin, trypsin, and glucosidase significantly decreased in the hepatopancreas of infected lobsters, as well as glycogen (Figure 7). In contrast, glucose and acilglycerides values increased in the plasma of infected samples. The differences were statistically significant and are consistent with the down-regulation observed in these enzymes at the proteomic level, as well as with our prediction of a general impairment in the capabilities of the hepatopancreas to metabolize triglycerides and glucose.

**FIGURE 8:**
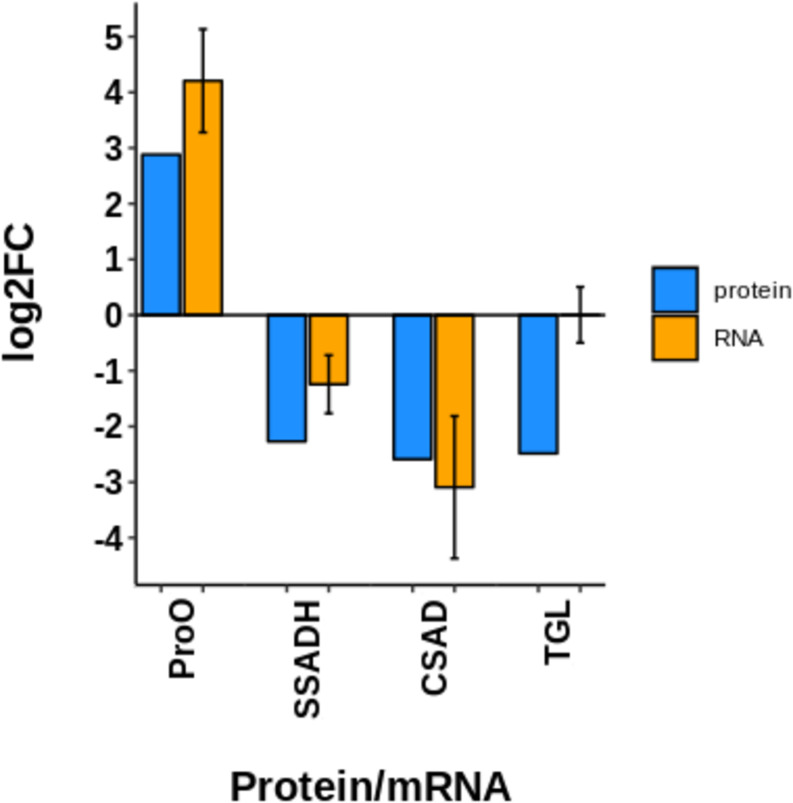
Comparison of the transcript expression of four selected genes with respect to its fold of change at proteomic level. ProPO: prophenol oxidase; SSADH: Succinate-semialdehyde dehydrogenase; CSAD: Cysteine sulfinic acid decarboxylase; TGL: triacylglycerol lipase. mRNA relative expression was normalized to a housekeeping gene (PyrC: Pyruvate carboxylase), their relative abundance was estimated by the ddCt method and values were log2 transformed. Expression data in general is according to proteomic data, except for TGL gene, which is probably subjected to post-transcriptional regulation.

Also, four proteins were randomly selected to complement the accuracy of the proteomics data using real-time qPCR. Gene expression patterns showed similar tendencies as those for protein expression, including: prophenol oxidase (proPO), succinate-semialdehyde dehydrogenase (SSADH), cysteine sulfinic acid decarboxylase (CSAD), and triacylglycerol lipase (TGL). mRNA relative expression was normalized to pyruvate carboxylase (PyrC) gene expression (Figure 8). The primer sequences for these genes are listed in Supplementary Table S4.

## Discussion

PaV1 causes a long-lasting infection with fatal consequences to juveniles *P. argus*.

In this work, we used a comparative proteomic strategy to analyze the alterations induced by PaV1 in the hepatopancreas in heavily infected juvenile lobsters. We analyzed the hepatopancreas because it is a key organ that regulates most of the physiological functions of lobsters, but it is also one of the main targets during PaV1 infection (Shields and Behringer, 2004; Li et al., 2008). This systemic infection initially affects fixed phagocytes in the hepatopancreas and surrounding connective tissues. Severely infected lobsters have a very deplorable nutritional condition, associated with physiological dysfunction due to a loss of digestive capacity. A marked atrophy in the hepatopancreas has been observed through histological analyses as well as a noticeable lack of or reduction of reserve inclusion cells (RI) and reduction of glycogen (Shields and Behringer, 2004; Behringer et al., 2011). Also, the significant differences in glucose, phosphates, cholesterol, triglycerides, proteins and lipases metabolites from hemolymph and tissues from infected and healthy juveniles of *P. argus*, support the hypothesis that their death results from metabolic depletion (Li et al., 2008; Herrera-Salvatierra et al., 2019).

Data provided herein can help to support this hypothesis and also would help to understand the origin of several aspects observed at macroscopic level in this host-pathogen interaction.

Here, alterations in the regulation of the enzymes involved in the energetic metabolism were detected. In the hepatopancreas of crustaceans, vacuoles with accumulation of glycogen and lipids are associated with active absorption of nutrients, whereas the mobilization of energetic reserves has been related to periods of starvation, molting, and reproduction (Sousa and Petriella, 2001). In the final stage of PaV1 infection, the glycogen reserves in the hepatopancreatic cells are dramatically reduced (Li et al., 2008; Pascual-Jiménez et al., 2012; Herrera-Salvatierra et al., 2019), and the results from this study confirmed such observations at a molecular level. In addition, the glucose values from the plasma of infected lobsters increased significantly with respect to healthy lobsters. Such increase of glucose in plasma might be either the direct consequence of the inability to metabolize and synthesize glycogen, or a reduced capability to metabolize glucose (Herrera-Salvatierra et al., 2019).

The general down-regulation of digestive proteases suggests an impairment in the capacity to metabolize proteins. The down-regulation of trypsin is of great importance, since this enzyme plays a central role in the maintenance of digestive capabilities of the lobster. Trypsin is one of the main proteases in the digestive tract of crustaceans (Perera et al., 2008), and is responsible for activating all the pancreatic enzymes by cleaving a short activation peptide from the amino-terminus of inactive zymogens (Gates and Travis, 1969; Sainz et al., 2006). The repression of this enzyme suggests a generalized disruption in the ability of the lobster to metabolize proteins, which constitute the principal component of its diet (Perera and Simon, 2015). Also, trypsin isoforms are responsible for other important physiological and immunological processes (Perera et al., 2012; Shi et al., 2009). Our results showed the up-regulation of prophenoloxidase (proPO), a key zymogen of the proPO activation cascade, an important enzymatic system that allows the encapsulation of pathogens (Amparyup et al., 2013). Recently, Herrera-Salvatierra et al. (2019), evaluated the induction of the activity of the proPO system in PaV1-infected spiny lobsters. Their results showed an increase in proPO, but a decrease in PO activity, which agrees with our results. This result implies that proPO activation mechanism mediated by proteolytic cleavage via the trypsin pathway is deactivated in infected lobsters. This is plausible since several families of proteases were identified as down-regulated.

In infected lobsters, PaV1 infection leads to accumulation of lipids in plasma, which apparently impart the milky appearance to the hemolymph (Pascual-Jiménez et al., 2012). In healthy organisms, the levels of triacylglycerides (TG) are enzymatically regulated by lipolysis, a process that implies a sequential enzymatic hydrolysis of TG, which is tightly regulated by triacylglycerol lipase (TGL). In this study, the downregulation of TGL could partially explain the inability of lobsters to metabolize triacylglycerides, resulting in their accumulation in the plasma. Moreover, the generalized deterioration of the metabolism of lipids represent a loss in the ability of lobsters to obtain energy from this source.

Histone proteins are involved in DNA packaging and regulation of DNA replication and transcription. In addition, histones or histone-derived peptides contribute to innate immune responses by different types of antimicrobial activity (Robinette et al., 1998; Richards et al., 2001; Fernandes et al., 2002). It was reported that histone H2B could mediate anti-virus immune defense reactions (Kobiyama et al., 2010; Arockiaraj et al., 2013). Interestingly, in shrimps infected with WSSV, H2A, H2B, H3A, and H4 proteins are up-regulated at transcriptional and proteomic levels (Patat et al., 2004; Hernández-Pérez et al., 2019).

It is probably that the generalized induction of proteins involved in genetic control and those involved in vesicular trafficking, and formation of phagolysosomes could be related to a hijack of PaV1 to complete its replicative cycle, assembly and translocation intracellularly. So, the analysis of the mechanisms that involve the intracellular trafficking of PaV1 is an interesting topic that requires further investigation, and preliminary data identified in this work can be a starting point.

In this study, we also observed a deregulation of important proteins involved in regulation of hormones and in the biosynthesis of neurotransmitters and neuromodulators. Based on these results we might hypothesize that PaV1 cause an endocrine disruption, since heavily infected lobsters exhibited a downregulation of both the estrogen sulfotransferase (SULT1E) and the hemolymph juvenile hormone (JHBP) binding proteins. Estrogen sulfotransferase can function as an effective modulator of local estrogen activity in target tissues (Song, 2010), by regulating the deactivation of estrogen by sulfation (Guo et al., 2016). Estrogen is not only a key hormone in reproduction, but its functions have also been implicated in the regulation of numerous processes including energy homeostasis.

In infected lobsters we also found several deregulated enzymes involved in the biosynthesis of gamma-aminobutyric acid (GABA), taurine and BH4. The alteration in the levels of such compounds could help to explain why heavily infected spiny lobsters become lethargic and incapable of rapid escape maneuvers, as has been recognized in other crustacean models (el Manira and Clarac, 2017). An increase in GABA content could partially explain the behavioral alterations in heavily PaV1-infected spiny lobster, but this needs further confirmation. On the other hand, a reduction in the content of sepiapterin reductase has a negative impact on the available BH4, and such reduction can produce dramatic alterations. For example, in humans, the deficiency of sepiapterin reductase (involved in the catalytic regeneration of BH4) provokes a progressive psychomotor retardation (Caccamo et al., 2013).

The cysteine sulfinic acid decarboxylase (CSAD) is another important enzyme, because it mediates the generation of taurine (Kerr et al., 2014). A repression of this enzyme could result in a reduction in the concentration of taurine in infected lobsters, as well as in the excretion of taurine and its biles salts. Due to the semi-open nature of the crustacean circulatory system, the change in the biosynthesis of this neuroactive compound could also affect the behavior and motility capabilities of the host (Giles and Usherwood, 1985; Stentiford et al., 1999). Considering that infected lobsters are avoided by healthy conspecifics (Behringer et al., 2011; Candia-Zulbarán et al., 2015), taurine could be tested as an odorant compound potentially mediating this social interaction (Giles and Usherwood, 1985).

**FIGURE 9:**
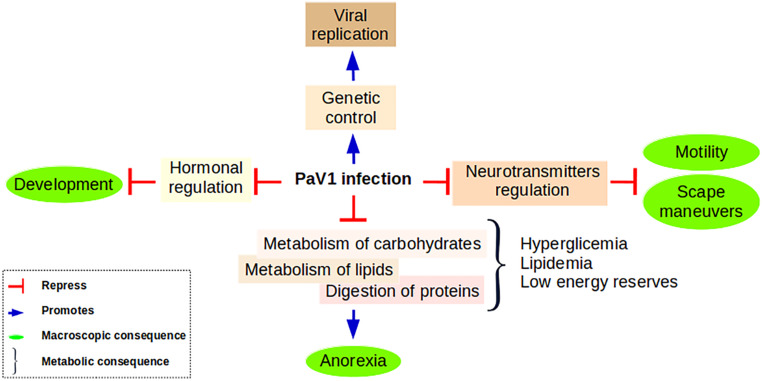
Proposed model summarizing the principal findings of this work. Anorexia is a consequence of the inability of a PaV1-infected lobster to digest its food due to its incapability to metabolize lipids and carbohydrates. These metabolites are accumulated in plasma, but in general the energy of the host is deficient. This low status associated with the deregulation of neurotransmitters and neuromodulators causes a significant reduction in the escape maneuvers and an important loss of motility. In addition, we propose that PaV1 infection causes a hormonal disruption that results in a development impairment reflected in the inability of the lobster for molting. PaV1 might take the control of the genetic machinery to promote its assembly: the overall up-regulation of the trafficking system could represent the way PaV1 is imported-exported throughout the cell.

Based on the above exposed information we propose a conceptual model to resume the consequences of PaV1 infection at the molecular level and its effects at a macroscopic level (Figure 9).

## Conclusion

This is the first comparative proteome profile of the hepatopancreas of *P. argus* spiny lobsters, showing the changes occurring at the molecular level in the hepatopancreas of *P. argus* in response to PaV1 infection that cause systemic alterations in its host. In heavily infected lobsters, PaV1 causes a depletion of proteins involved in immunological, energy, and nutritional functions. In addition, some key enzymes involved in neuromodulation were unbalanced. In general, findings from this study will help to explain at the molecular level why lobsters become lethargic and develop anorexia in the final stage of the viral infection. Some initial keys provided here can be used to make a comprehensive model of the action modes of PaV1.

## Data Availability Statement

The mass spectrometry proteomics data have been deposited to the ProteomeXchange Consortium (http://proteomecentral.proteomexchange.org) via the iProX partner repository with the dataset identifier PXD018882.

## Author Contributions

JZ-B, RR-C, and EL-Á conceptualized the study. JZ-B and RR-C designed the experiments. JZ-B, ER-M, and JE-C analyzed the proteomic and contributed to the data curation. JZ-B, RR-C, EL-Á, AH-P, PB-F, ER-M, NH-S, and CP-J wrote and edited the manuscript. JZ-B, PB-F, EL-Á, and IH-V contributed to the sampling field coordination. RR-C and EL-Á contributed to the project administration. JZ-B contributed to the formal analysis. RR-C, EL-Á, and PB-F contributed to the funding acquisition. JZ-B, IH-V, AF-G, NH-S, and CP-J contributed to the methodology. RR-C and EL-Á contributed to the supervision. JZ-B, IH-V, and AF-G contributed to the visualization.

## Conflict of Interest

The authors declare that the research was conducted in the absence of any commercial or financial relationships that could be construed as a potential conflict of interest.

## Footnotes

1 http://transdecoder.github.io

## Supplementary Material

FIGURE S1 Tree-map of the GO annotation of the proteome identified at the molecular function level. Process related to lipid metabolism, protein synthesis and proteolysis, immune system and carbohydrate metabolism are well represented in accordance with the physiological function of the hepatopancreas. Proteins were annotated with InterProScan5 and their frequencies GO were exported to Revigo.

FIGURE S2 Heatmap of the diferential proteins and Pearson’s correlation analysis of the global protein profile per condition. **(A)** Heatmap plot of the regulated proteins. Original values were ln(x + 1)-transformed. Rows centered; no scaling is applied to rows. Rows were clustered using correlation distance and Ward linkage. Pearson’s correlation analysis of the abundances of total proteins for healthy and **(B)** and infected **(C)** lobsters.

FIGURE S3 Down-regulated enzymes involved in glycogen and glucose metabolism. The control of the synthesis and catabolism of glycogen is blocked. Down-regulated enzymes: UTP-glucose-1-phosphate uridylyltransferase [EC:2.7.7.9], endoglucanase [EC:3.2.1.4] and glycogen phosphorylase [EC:2.4.1.1], and fructose-bisphosphate aldolase, class I [EC:4.1.2.13].

FIGURE S4 Deregulated enzymes involved in lipid metabolism. A general down-regulation in pathways that control the degradation, and synthesis of lipids is observed. Down regulated enzymes (red): enoyl-CoA hydratase/long-chain 3-hydroxyacyl-CoA dehydrogenase [EC:4.2.1.17 1.1.1.211], enoyl-CoA hydratase [EC:4.2.1.17], acyl-CoA oxidase [EC:1.3.3.6], enoyl-CoA hydratase [EC:4.2.1.17], acyl-coenzyme A thioesterase 1/2/4 [EC:3.1.2.2], aldehyde dehydrogenase (NAD+) [EC:1.2.1.3], triacylglycerol lipase [EC:3.1.1.3], 2-hydroxyacyl-CoA lyase 1 (HPCL2). Up-regulated enzyme (blue): 3-hydroxyacyl-CoA dehydrogenase [EC:1.1.1.35].

FIGURE S5 Metabolism of tetrahydrobiopterin (BH4). Down-regulated enzyme (red): sepiapterin reductase [EC:1.1.1.153]. Sepiapterin reductase catalyzes the final step in BH4 synthesis, which is a cofactor of the three aromatic amino acid hydroxylase enzymes. It is also a cofactor for the production of nitric oxide (NO), and is a cofactor of ether lipid oxidase.

FIGURE S6 Deregulation of the metabolism of taurine. Down_regulated enzyme (red): sulfinoalanine decarboxylase [EC:4.1.1.29]. This enzyme is responsible of the synthesis of taurine.

FIGURE S7 Down regulation of enzymes implicated in the synthesis of neurotransmitters. Down regulated enzymes (red): 4-aminobutyrate aminotransferase/(S)-3-amino-2-methylpropionate transaminase [EC:2.6.1.19 2.6.1.22], succinate-semialdehyde dehydrogenase [EC:1.2.1.24], and glutamine synthetase [EC:6.3.1.2].

TABLE S1 Evidence data of the proteome of the hepatopancreas of *P. argus* with the annotations and characteristics of the identified proteins.

TABLE S2 Complete list of significantly down-regulated proteins. Proteins with a log2 fold change (FC) ≤ −1.5 and a *p*-value < 0.05 were considered as differentially regulated.

TABLE S3 Complete list of significantly up-regulated proteins. Proteins with a log2 fold change (FC) ≥ 1.5 and a *p*-value < 0.05 were considered as differentially regulated.

TABLE S4 List of primers used to validate the proteomic data.
