# Supplementary figures and images for: iTRAQ-Based Proteomic Profile Analysis of the Hepatopancreas of Caribbean Spiny Lobsters Infected With Panulirus argus Virus 1: Metabolic and Physiological Implications
Source: Front Microbiol. 2020 May 29;11:1084. doi: 10.3389/fmicb.2020.01084 (PMC7273172; doi:10.3389/fmicb.2020.01084)

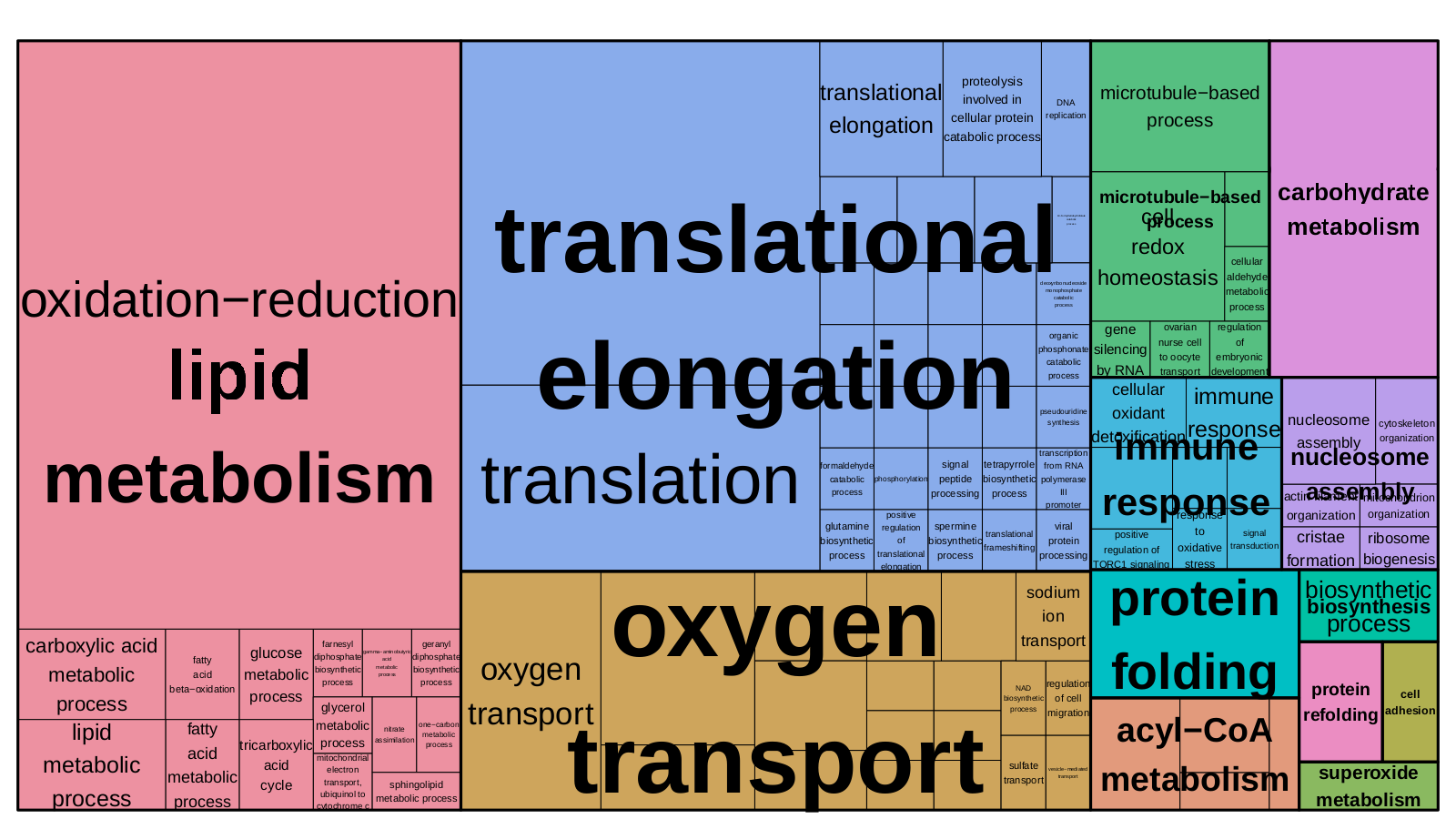

Supplement: FIGURE S1 — Tree-map of the GO annotation of the proteome identified at the molecular function level. Process related to lipid metabolism, protein synthesis and proteolysis, immune system and carbohydrate metabolism are well represented in accordance with the physiological function of the hepatopancreas. Proteins were annotated with InterProScan5 and their frequencies GO were exported to Revigo. [file Image_1.TIFF]

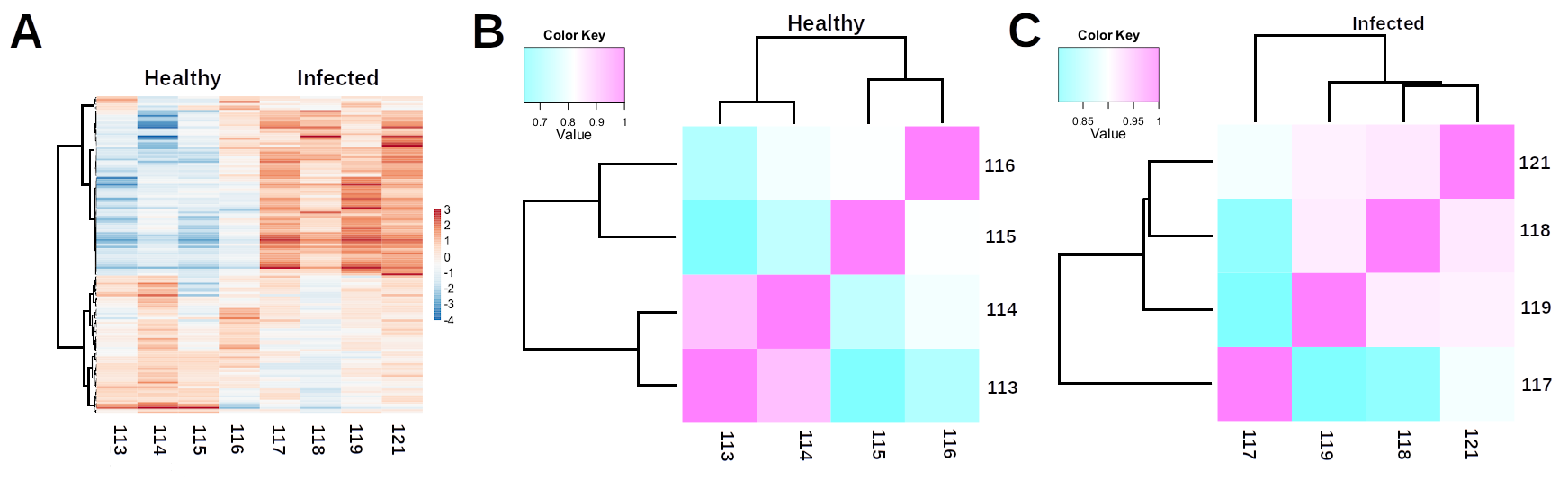

Supplement: FIGURE S2 — Heatmap of the diferential proteins and Pearson’s correlation analysis of the global protein profile per condition. (A) Heatmap plot of the regulated proteins. Original values were ln(x + 1)-transformed. Rows centered; no scaling is applied to rows. Rows were clustered using correlation distance and Ward linkage. Pearson’s correlation analysis of the abundances of total proteins for healthy and (B) and infected (C) lobsters. [file Image_2.TIFF]

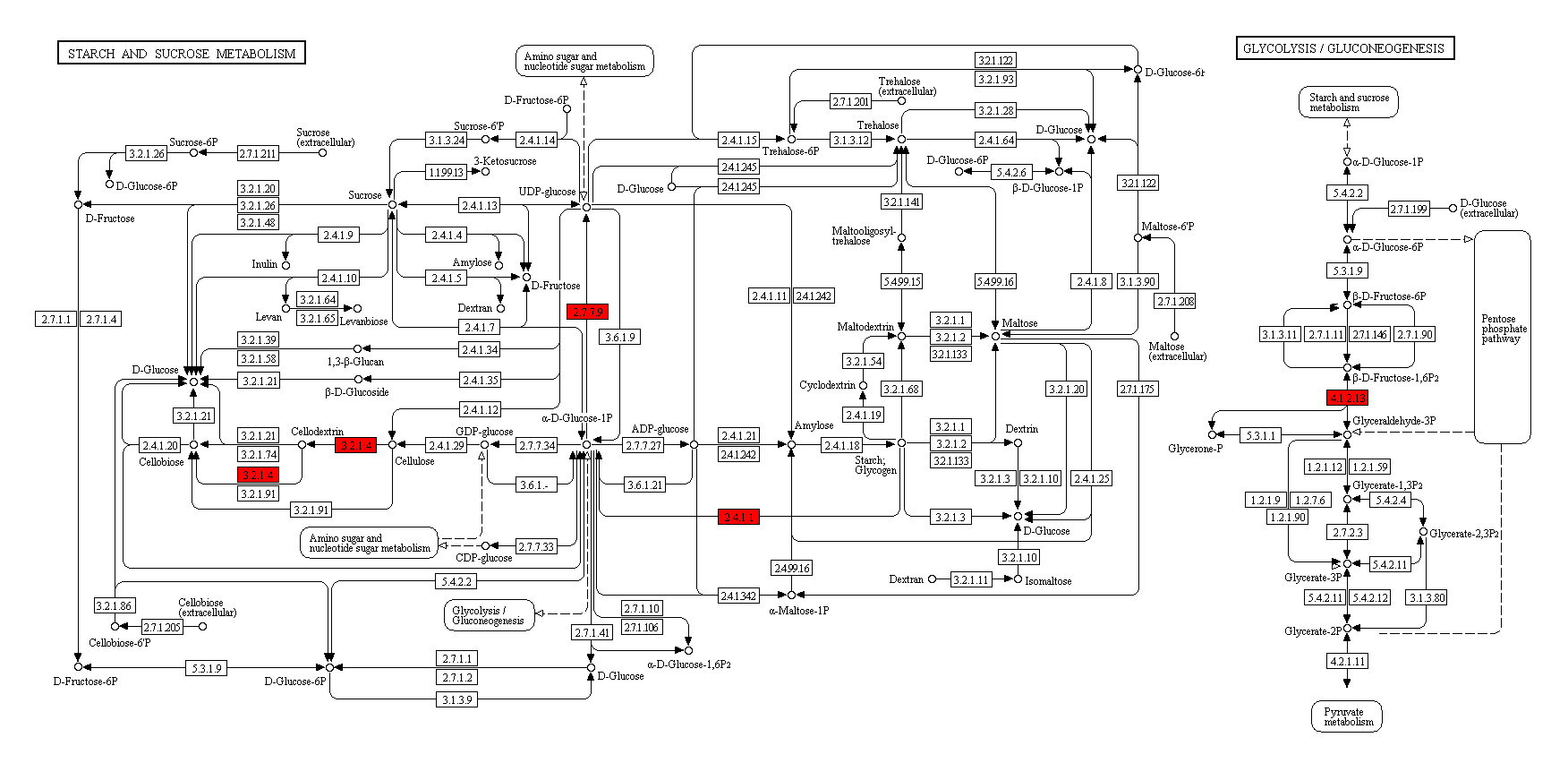

Supplement: FIGURE S3 — Down-regulated enzymes involved in glycogen and glucose metabolism. The control of the synthesis and catabolism of glycogen is blocked. Down-regulated enzymes: UTP-glucose-1-phosphate uridylyltransferase [EC:2.7.7.9], endoglucanase [EC:3.2.1.4] and glycogen phosphorylase [EC:2.4.1.1], and fructose-bisphosphate aldolase, class I [EC:4.1.2.13]. [file Image_3.TIFF]

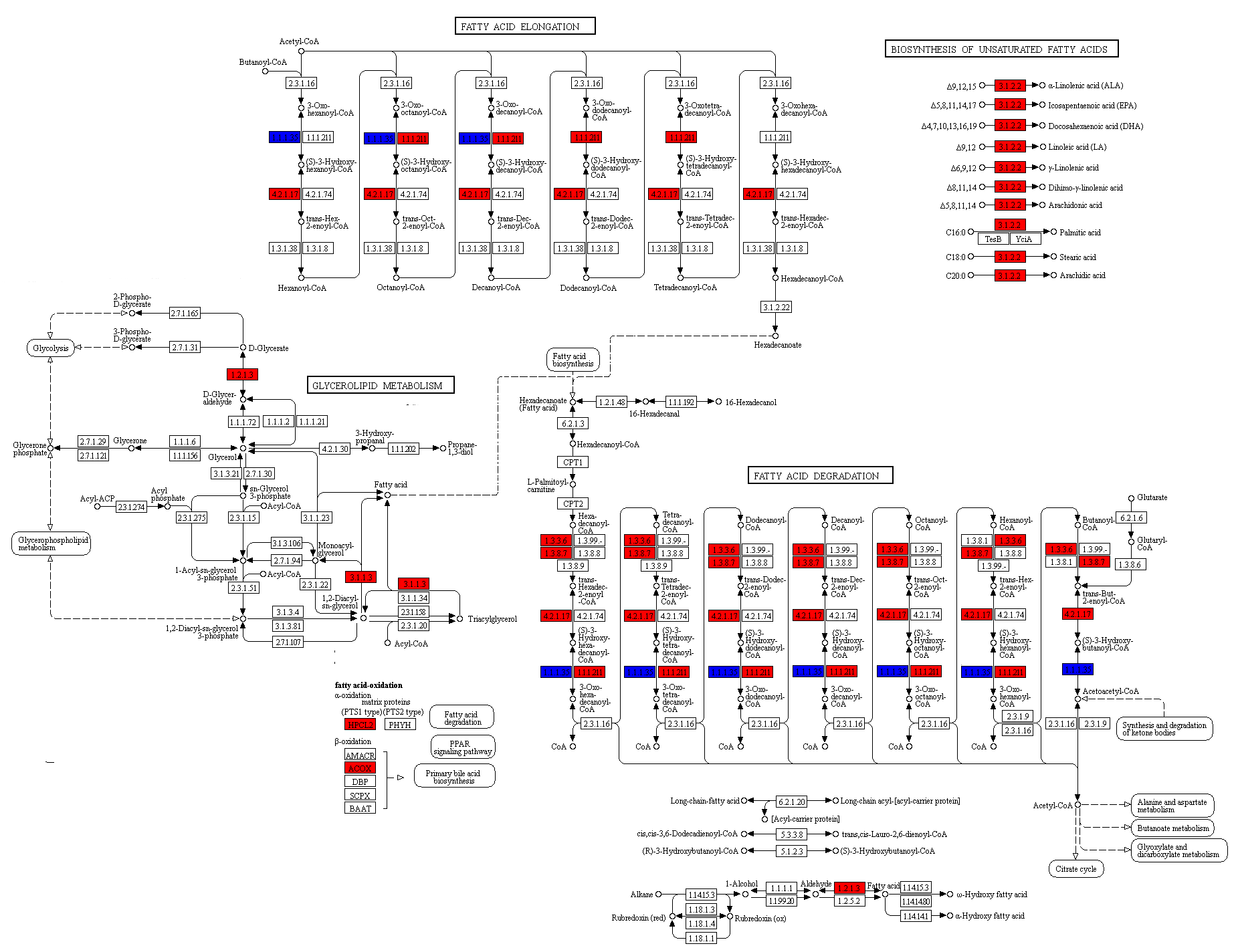

Supplement: FIGURE S4 — Deregulated enzymes involved in lipid metabolism. A general down-regulation in pathways that control the degradation, and synthesis of lipids is observed. Down regulated enzymes (red): enoyl-CoA hydratase/long-chain 3-hydroxyacyl-CoA dehydrogenase [EC:4.2.1.17 1.1.1.211], enoyl-CoA hydratase [EC:4.2.1.17], acyl-CoA oxidase [EC:1.3.3.6], enoyl-CoA hydratase [EC:4.2.1.17], acyl-coenzyme A thioesterase 1/2/4 [EC:3.1.2.2], aldehyde dehydrogenase (NAD+) [EC:1.2.1.3], triacylglycerol lipase [EC:3.1.1.3], 2-hydroxyacyl-CoA lyase 1 (HPCL2). Up-regulated enzyme (blue): 3-hydroxyacyl-CoA dehydrogenase [EC:1.1.1.35]. [file Image_4.TIFF]

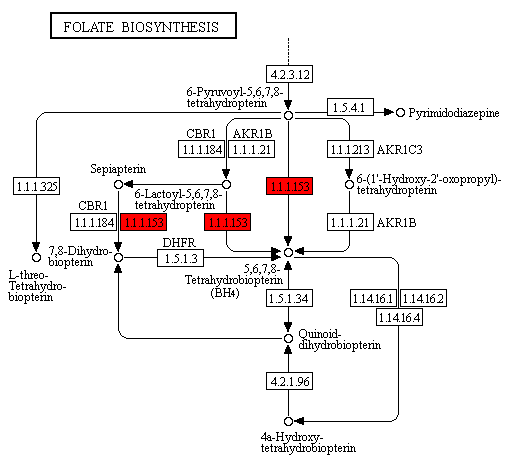

Supplement: FIGURE S5 — Metabolism of tetrahydrobiopterin (BH4). Down-regulated enzyme (red): sepiapterin reductase [EC:1.1.1.153]. Sepiapterin reductase catalyzes the final step in BH4 synthesis, which is a cofactor of the three aromatic amino acid hydroxylase enzymes. It is also a cofactor for the production of nitric oxide (NO), and is a cofactor of ether lipid oxidase. [file Image_5.TIFF]

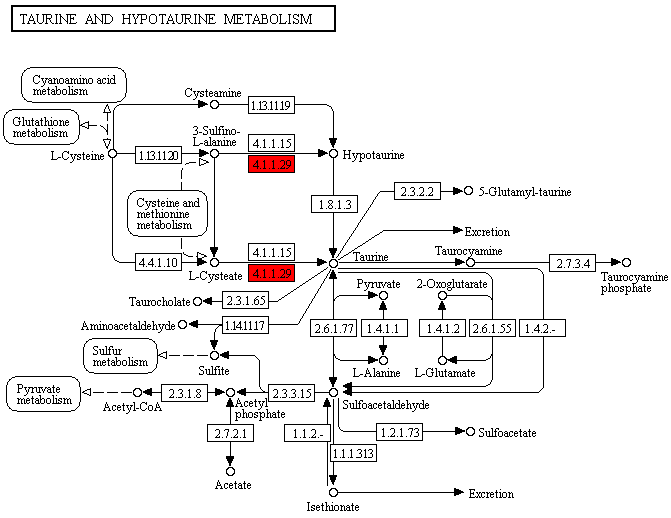

Supplement: FIGURE S6 — Deregulation of the metabolism of taurine. Down_regulated enzyme (red): sulfinoalanine decarboxylase [EC:4.1.1.29]. This enzyme is responsible of the synthesis of taurine. [file Image_6.TIFF]

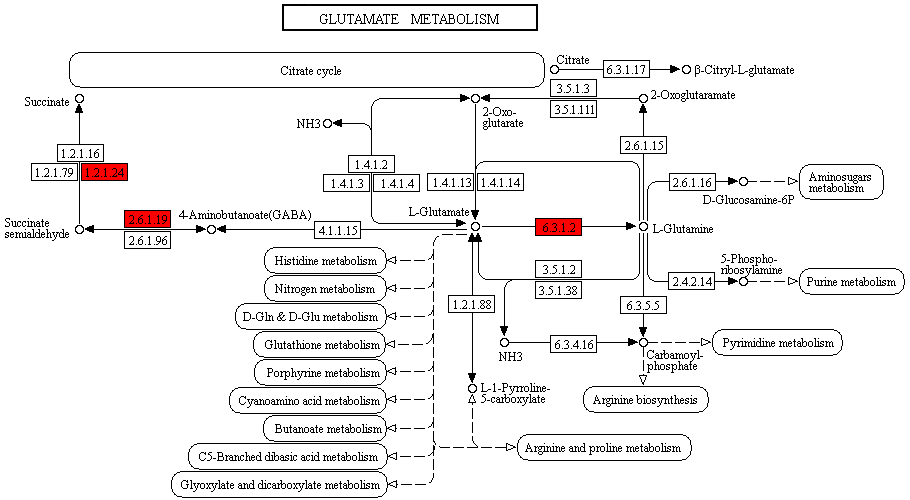

Supplement: FIGURE S7 — Down regulation of enzymes implicated in the synthesis of neurotransmitters. Down regulated enzymes (red): 4-aminobutyrate aminotransferase/(S)-3-amino-2-methylpropionate transaminase [EC:2.6.1.19 2.6.1.22], succinate-semialdehyde dehydrogenase [EC:1.2.1.24], and glutamine synthetase [EC:6.3.1.2]. [file Image_7.TIFF]
